# Performance of novel MgS doped cellulose nanofibres for Cd(II) removal from industrial effluent – mechanism and optimization

**DOI:** 10.1038/s41598-019-49076-2

**Published:** 2019-09-02

**Authors:** Nalini Sankararamakrishnan, Rishabh Singh, Ila Srivastava

**Affiliations:** 1 Centre for Environmental Science and Engineering, Indian Institute of Technology Kanpur, Kanpur, U. P. 208016 India; 2 Department of Environmental Science and Technology, Banaras Hindu University, Varanasi, U.P. 221005 India

**Keywords:** Environmental chemistry, Environmental sciences, Pollution remediation

## Abstract

Green environment friendly and novel nano MgS decorated cellulose nanofibres (MgS@CNF) were prepared, characterized and evaluated towards the removal of heavy metal namely, cadmium from aqueous solutions. Cellulose nanofibres acted as a template for effective dispersion of MgS nanoparticles and also aid in the complexation of cadmium ions. In depth X-ray photoelectron spectroscopy (XPS), X-ray diffraction (XRD) and Fourier transform infra red spectroscopy (FTIR) studies revealed that doped MgS on mild acidification yields *insitu* production of H_2_S which effectively complexes cadmium ion to form cadmium sulfide. The reaction followed pseudo first order kinetics with regression coefficient in the order of 0.98. A very high Langmuir adsorption capacity in the order of 333.33 mg/g was obtained for MgS@CNF. Finally, MgS@CNF was applied towards the removal of cadmium from organic and TDS rich tannery waste water. MgS@CNF was effective in bringing down the concentration from ppm to ppb levels.

## Introduction

Heavy metal pollution by industrial wastewater is one of the indispensable environmental challenges worldwide. Among various heavy metals, non-biodegradability and bioaccumulation makes Cadmium (Cd) as one of the most toxic heavy metals^1^. Cadmium primarily finds use in plastics and steel production and it has been listed as Group-B1 and category-I carcinogen by the US EPA and International Agency for Research on Cancer respectively^2^. Mainly Cd accumulates in bones, pancreas, livers and kidneys, and, which causes various ailments like nephritis, neuralgia, hypertension, secretion disorder and anemia^3^. Various methods have been reported for removal including chemical precipitation, reverse osmosis, adsorption, and ion-exchange^4^. Among these methods, removal of heavy metal contamination by adsorption process is considered as economic, reliable, and effective method^5,6^. Various adsorbents like activated carbon^7^, zeolites^8^, nanozerovalent iron^9,10^, carbon nano tubes^11,12^, graphene^13^, functionalized chitosan^14,15^, low cost agricultural wastes^16,17^ have been found useful for its removal. It is to be noted among these, adsorbents modified with sulfur containing ligands exhibited excellent adsorption capacity owing to soft-soft interaction effective complexation of Cd and sulphur ligands^15,18,19^. Further it was also observed that nanocomposites with increased surface area provided efficient metal ion binding^20,21^. Thus in this work green biodegradable cellulose nanofibers were extracted from low cost agricultural waste sugarcane bagasse and nano MgS were effectively doped on this nanofiber and evaluated for its capacity towards cadmium. This is the first time we have reported a novel MgS doped bio-nanocomposite. Cellulose nanofiber provides a template for effective dispersion of MgS and also helps in preventing the agglomeration of the nano MgS. Characterization with various analytical techniques, optimization, mechanism of interaction and its application towards cadmium removal from tannery waste water are detailed in the following sections.

## Results and Discussion

### Characterization of MgS@CNF and mechanism of interaction

**Figure 1:**
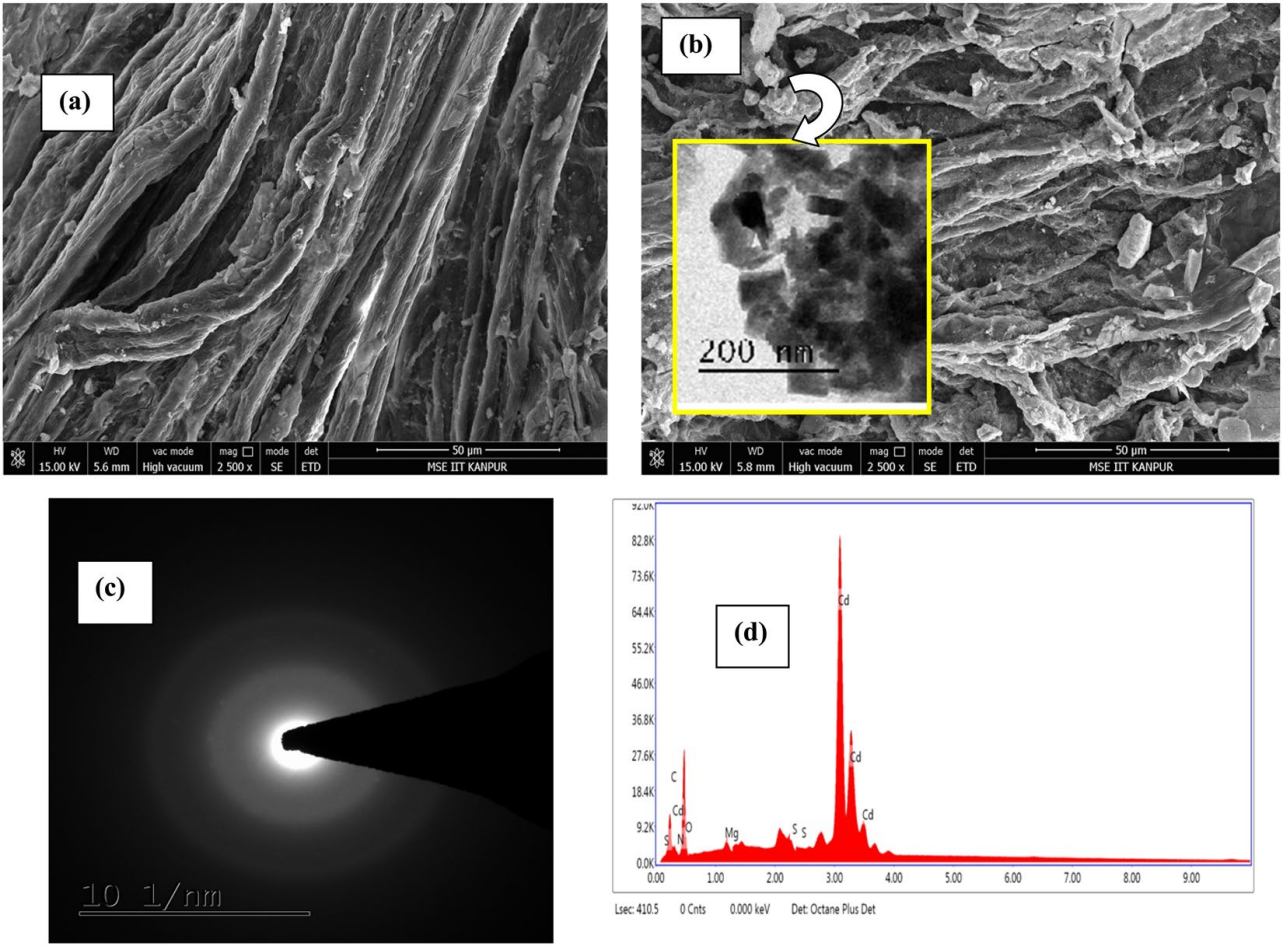
(**a**) SEM image of CNF (**b**). SEM image of Cd(II) loaded MgS@CNF inset TEM image of MgS (**c**). SAED pattern of MgS@CNF (**d**). EDAX plot of Cd-MgS@CNF.

**Figure 2:**
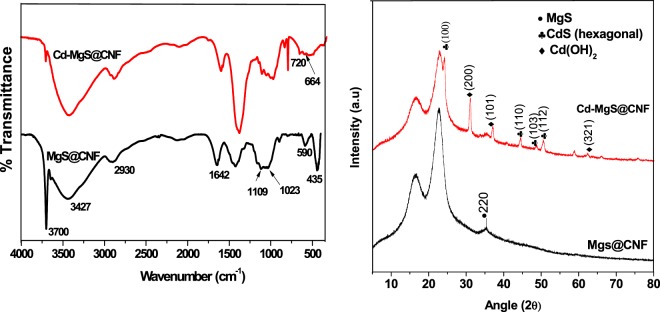
(**a**) FTIR spectra of MgS@CNF and Cd-MgS@CNF (**b**). XRD spectra of MgS@CNF and Cd-MgS@CNF.

The scanning electroscopy images of MgS@CNF and Cd loaded MgS@CNF are shown in Fig. 1a,b respectively. It is evident MgS is uniformly loaded on nanofibers and adsorption of Cd on the surface of the fibers is also clear. Further EDX spectra revealed the presence of Mg, S and Cd on Cd loaded MgS@CNF (Fig. 1c). The FTIR spectra MgS@CNF are shown in Fig. 2a. Broad band around 3700 cm^−1^ in both MgS@CNF and Cd- MgS@CNF could be attributed to –OH stretching vibrations of hydrogen bonded cellulose moeity whereas the sharp peak around 3427 cm^−1^ is due to the free –OH stretching vibrations. The stretching bands of O-CH_3_ are observed at 2930 cm^−1^ which arises from the trace lignin of cellulose fibres^22,23^. The S-O group gave rise symmetric and asymmetric vibrations at 1109 and 1023 cm^−1^ respectively^24^. Very sharp and strong bands are observed in both the spectra at 1642 cm^−1^ which could be attributed to non dissociated water molecules^25^. In MgS@CNF anchoring of sulfur molecules on cellulose nanofibers is proved by the presence of vibrations at 435 cm^−1^ and 590 cm^−1^ which is due to presence of polysulfide and disulfide respectively^24^. After Cd(II) adsorption, appearance of Cd-S stretching vibrations were observed at 664 and 720 cm^−1^ ^26^ confirming the formation of CdS on the surface of MgS@CNF. The XRD pattern of MgS@CNF and Cd(II) loaded MgS@CNF were recorded and the diffraction pattern is shown in Fig. 2b. Sharp peaks were absent in MgS@CNF due to the amorphous nature of MgS. However, cadmium loaded MgS@CNF exhibited peaks indexed to Cd(OH)_2_ with strong (101), (200) and (321) reflections (JCPDS card No. 31-0118) and hexagonal CdS with strong reflections of miller indices (100), (110), (103) and (112) which are consistent with the literature values(JCPDS card No. 41-1049).

**Figure 3:**
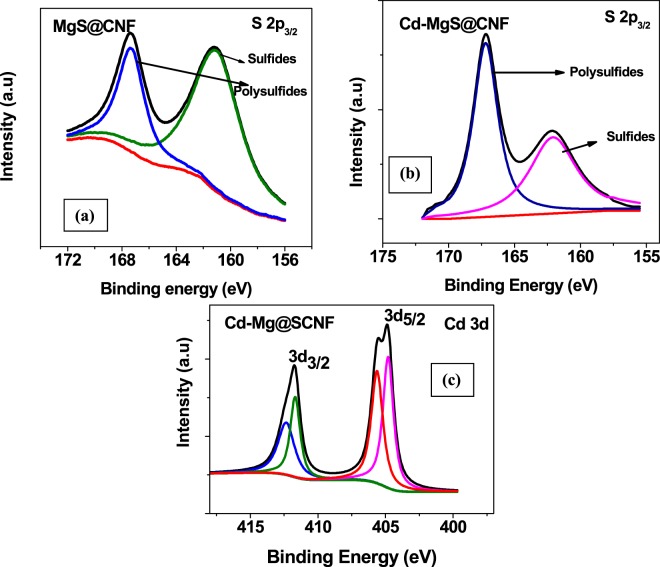
XPS molecular level spectra of S 2p in MgS@CNF (**a**). before and (**b**). after Cd(II) loading (**c**). Molecular level spectra of Cd 3d in Cd-MgS@CNF.

XPS survey spectra of cadmium loaded MgS@CNF exhibited peaks corresponding to sulfur, Mg and Cd indicating effective loading of magnesium and complexing of cadmium with sulfur ions (Fig. S1, Supporting Information). Furthermore the deconvoluted core level XPS spectra of S 2p before and after loading with Cd are shown in Fig. 3a,b respectively. As expected, before Cd loading all the sulfur ions were either as monosulfides^27^ or polysulfides^28^ corresponding to binding energies 161.01 and 167.31 eV respectively. However after Cadmium loading the binding energies of S2p_3/2_ are shifted to lower binding energy values. The peak at binding energy value 160.68 ev correspond to CdS^29^ and the strong peak at 166.0 ev corresponds to polysulfides of Cd/Mg ions. Figure (3c) depicts Cd-3d spectra, and the details are given in Table 1. It is evident that peaks were observed at 405.6 eV and 412.3 eV corresponding to 3d5/2 and 3d3/2 respectively. The binding energies obtained were consistent with that of CdS^30,31^ and in addition to these peaks, additional peaks of at 405.5 eV for 3d5/2 and 411.8 eV for 3d3/2 were obtained. These peak positions corresponds to cadmium hydroxide^32,33^. Thus from the afore mentioned discussions, following mechanism is postulated

1 $$2{\rm{MgS}}+4{{\rm{H}}}_{2}{\rm{O}}+2{{\rm{H}}}^{+}\to 2{\rm{Mg}}{({\rm{OH}})}_{2}+2{{\rm{H}}}_{2}{\rm{S}}$$

2 $$2{{\rm{MgS}}}_{2}+4{{\rm{H}}}_{2}{\rm{O}}+4{{\rm{H}}}^{+}\to 2{\rm{Mg}}{({\rm{OH}})}_{2}+4{{\rm{H}}}_{2}{\rm{S}}$$

3 $${\rm{Cd}}({\rm{II}})+{{\rm{H}}}_{2}{\rm{S}}\to {\rm{CdS}}$$

**Table 1:** Molecular level XPS analysis of MgS@CNF before and after Cd(II) loading.

Before/After Cd(II) Loading	Element	B.E. (eV)	Fraction (%)	Species	Reference
Before	Sp3/2	161.10 167.31	70.3 29.7	S^2−^ Polysulfides of Mg	^27, 28^
After	Sp3/2	160.68 166.00	53.5 46.5	CdS Polysulfides of Mg/Cd	^29, 28^
After	Cd d5/2	412.3 411.6	19.8 18.3	CdS Cd(OH)_2_	^30– 33^
	Cd d1/2	405.6 404.8	31.9 30.0	CdS Cd(OH)_2_	^30– 33^

Apart from CdS, it is evident from XPS study that hydroxide salts of Cadmium are also observed which could be attributed to the interaction between the hydroxide groups of cellulose moiety with Cd(II) ions. Further, it is worth mentioning that EDX studies around 2.1 weight % of Sulfur is present on the adsorbent. Since we use low amount of adsorbent, the amount of hydrogen sulfide gas released is sufficient enough to remove the pollutant and will not pose a secondary pollution.

### Effect of Initial pH on adsorption of Cd by MgS@CNF

Initial solution pH plays a significant role in the adsorption of Cd by MgS@CNF and CNF. There are two processes involved in the adsorption of Cd by MgS@CNF. Initially, MgS/MgS_2_ undergoes acid hydrolysis to yield *insitu* H_2_S gas as depicted in equation 1 and 2. Later, produced H_2_S gas reacts with Cd(II) ions to form CdS as shown in equation 3. Thus at low pH though formation of H_2_S is favorable it is ineffective in complexing with Cd ions. At very high pH values though formation of CdS is favorable, however, formation of H_2_S is insignificant. Hence between pH 5.5 to 6.0 both the processes namely evolution of H_2_S and formation of CdS is favorable.

**Figure 4:**
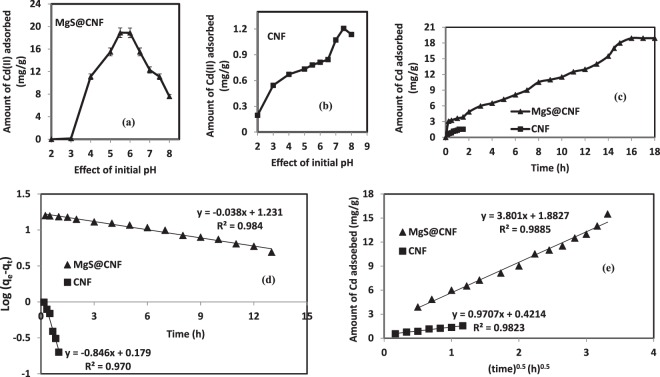
Effect of Initial pH on Cd(II) Adsorption by (**a**). MgS@CNF (**b**). CNF (**c**). Kinetics of Cd(II) Adsorption on CNF and MgS@CNF (**d**). Pseudo First order kinetics plot and (**e**). Web-morris plot of CNF and MgS@CNF and cadmium systems.

Thus from the Fig. 4a it is evident that adsorption is negligible at pH values less than 3 and then slowly it increases with increase in pH. Cadmium removal increased from 77.5% to 94.5% with increase of pH from 5 to 5.5 and remained constant till pH 6 and further increase in pH to 8 resulted in decreased adsorption of around 40%. In the case of CNF, it is evident that adsorption of Cd increases with pH and reaches a maximum of 10.72% at pH 7.5 and subsequent increase in pH resulted in decreased adsorption of Cd ions by CNF. This could be attributed to the complexing of OH ions of cellulose and cadmium ions to form cadmium hydroxide. At higher pH values, cadmium exists as Cd(OH)_2_ and precipitation occurs and free Cd ions are unavailable for complexation.

### Reaction kinetics of Cd with CNF and MgS@CNF

The reaction kinetics Cd removal by CNF and MgS@CNF indicated a rapid binding of Cd ions by the nanocomposite during the initial minutes followed by a gradual increase until a steady state of equilibrium was reached. Steady state was achieved after 1 h and 16 h of equilibration for CNF and MgS@CNF respectively (Fig. 4b). In the case of MgS@CNF slow release of H_2_S took place and hence it took longer time to achieve equilibrium compared of plain CNF. Similar to this study longer equilibration times have been reported on the removal of Cd by chitin^34^, xanthated chitosan^35^. Obtained data was modeled by pseudo first order equation given below:

4 $$\mathrm{log}({q}_{e}-{q}_{t})=\,\mathrm{log}({q}_{e})-\frac{{k}_{1}}{2.303}T$$

where q_e_ and q_t_ are the amount of Cd ions adsorbed by MgS@CNF in mg/g at given time “T” and equilibrium respectively and *k*_1_ is the pseudo first order constant in h^−1^. Various constants obtained are detailed in Table 2 and the plot is depicted in Fig. 4c. Adsorption of pollutants onto onto solid surface is also governed by intraparticle diffusion. Thus intraparticle diffusion rates of Cd ions onto CNF and MgS@CNF could be modeled using Weber and Morris model given by equation (5)

5 $${q}_{t}={K}_{int}\sqrt{{\rm{t}}}$$

where “*q*_*t*_” is the amount of Cd adsorbed by CNF/MgS@CNF at a given time “t” and “*K*_int_” is the intraparticle diffusion constant. Both CNF and MgS@CNF plots of qt *Vs* T yielded straight line with a high correlation coefficient and intercept Fig. (4d). Thus it could be concluded that the intraparticle diffusion of Cd ions onto CNF/MgS@CNF is not the sole rate determining step governing sorption.

**Table 2:** Kinetic and isotherm parameters on adsorption of Cadmium by CNF and Mgs@CNF.

Adsorbent (pH)	Isotherm Constants						Kinetics Constants				
	Langmuir			Freundlich			Pseudo First Order			Web Morris	
	Q(max) (mg/g)	b	R^2^	1/n	K_f_	R^2^	*k*_*L*_ (h^−1^)	*q*_*e*_ (mg/g)	R^2^	*k*_*int*_ (g/mg/h^0.5^)	R^2^
CNF (7.5)	7.81 ± 0.56	0.2415	0.99	3.559	2.576	0.911	0.0322	1.51	0.97	0.970	0.98
MgS@CNF (5.5)	333.33 ± 2.02	0.1071	0.99	1.280	29.785	0.989	0.0875	17.02	0.98	3.801	0.99

### Adsorption isotherms of Cd with CNF and MgS@CNF systems

**Figure 5:**
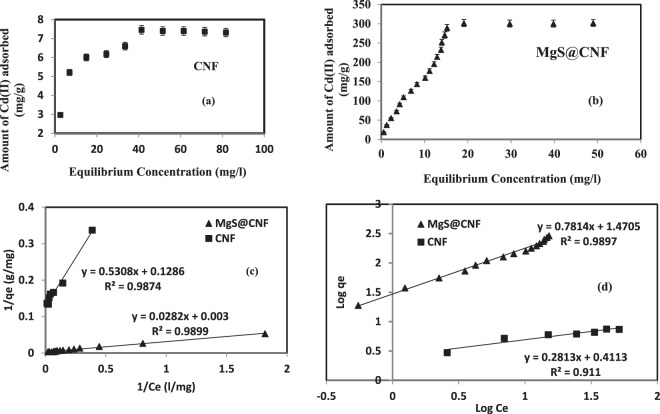
Equilibrium Adsorption isotherm (**a**) CNF (**b**). MgS@CNF, (**c**). Langmuir Plot and (**d**). Freundlich Plots of CNF and MgS@CNF and cadmium systems.

Cadmium adsorption equilibrium isotherms for CNF and MgS@CNF are shown in Fig. 5a. Obtained adsorption data using CNF and MgS@CNF were modeled using widely used Langmuir and Freundlich isotherm models. Unlike Freundlich model, Langmuir model is based on the assumption of monolayer and the both the models are represented by equations 6 and 7 respectively.

6 $$\mathrm{log}\,{q}_{e}=\frac{1}{n}\,\mathrm{log}\,{c}_{e}+\,\mathrm{log}\,{k}_{f}$$

7 $$\frac{1}{{q}_{e}}=\frac{1}{{c}_{e}{q}_{\max b}}+\frac{1}{{q}_{max}}$$

where q_e_ and c_e_ are the amount of cadmium adsorbed (mg/g) and concentration of cadmium at equilibrium (mg/l) respectively. “K_f_” and “n” are freundlich adsorption and affinity constants. “q_max_” and “b” are Langmuir monolayer adsorption capacity (mg/g) and equilibrium constant (ml/mg) respectively. Langmuir and Freundlich plots are presented in Fig. 5b,c respectively and obtained adsorption constants are listed in Table 2. A comparison on the adsorption capacities of various sorbents recently reported in literature is listed in Table S1 of supporting information. Adsorbents anchored with sulfur containing groups are given for comparison purposes. It is evident from the data thatadsorption capacity of the MgS@CNF towards Cd(II) is higher than the ones reported in the literature. This could be attributed to the insitu generation of hydrogen sulfide gas which effectively complexes with Cd(II).

### Thermodynamic studies

Thermodynamic parameters including change in Gibbs free energy (ΔG°), enthalpy (ΔH°) and entropy (ΔS°)were calculated by conducting the adsorption experiments of MgS@CNF and Cd(II) systems at three different temperatures namely 25, 35 and 45 °C. From adsorption data, thermodynamic parameters were determined. Initially, *Kc*, the equilibrium constant for MgS@CNF and Cd(II) was determined using equation (8)

8 $${K}_{C}=\frac{{C}_{A}}{{C}_{e}}$$

where, C_A_(g/L) is the concentration of Cd(II) in the aqueous phase and C_e_ is the equilibrium concentration of Cd(II) (g/l). Then, equation (9) was used calculate Gibbs free energy change change.

9 $${\rm{\Delta }}{G}^{\circ }=-\,RT\,\mathrm{ln}\,Kc$$

where R is the gas constant, T is the temperature in Kelvin. Further, Van’t Hoff equation (10) was used to calculate entropy (ΔS) and enthalpy change (ΔH) was determined:

10 $$\mathrm{log}\,Kc=\frac{{\rm{\Delta }}{S}^{\circ }}{2.303}-\frac{{\rm{\Delta }}{H}^{\circ }}{2.303RT}$$

A linear plot was constructed between ln *K*_*c*_ vs. 1/T of Cd(II) and MgS@CNF, and ΔS° and ΔH° were determined from the intercept and slope, respectively. The results obtained are presented in Table S2 of the supporting information. The spontaneity and feasibility of the adsorption process between MgS@CNF and Cd(II) ions is indicated by negative free energy values, while the positive ΔH° values indicate the endothermic nature of the sorption process. Entropy (Δ*S*°) of adsorption yielded positive values which could be attributed to Cd(II) ion dehydration during surface sorption onto MgS@CNF.

### Application to real industrial effluent

Industrial effluent from local tanneries was collected and the characteristics of the same are listed in Table S2 of the supporting information. Tannery effluents were treated with 0.1 g of MgS@CNF after 10 times dilution and adjusting the pH 5.5. The solutions were equilibrated for 16 h and the concentration of cadmium and other heavy metal ions were determined by ICPMS. It is evident from the data (Table 3) that MgS@CNF is effective in removing the cadmium concentration from ppm to ppb levels. Apart from cadmium, it is also evident that it is efficient in bringing down the concentration other metal ions including Cr, Pb, As, Zn and COD as well. Insitu generation of H_2_S during adsorption promotes the precipitation of other soft metals like Zn, Pb, As and Zn onto the surface of the adsorbent and thus we observe the reduction in concentration of these metal ions. Tannery effluent comprises both Cr(VI) and Cr(III) species. During adsorption, Cr(VI) could be reduced to Cr(III) by the sulfide ions. Reduced Cr(III) and Cr(III) already present in tannery effluent could be immobilized as hydroxides/sulfides onto the adsorbent surface. Because of this redox reaction a decreased value of chemical oxygen demand is observed after adsorption. Hence the prepared adsorbent could be applicable to treat other wastewaters as well.

**Table 3:** Application of MgS@CNF to Industrial effluent.

Effluent	Before Adsorption (mg/l)						After Adsorption (mg/l)					
	Cd	Total Cr	Pb	Zn	As	COD	Cd	Total Cr	Pb	Zn	As	COD
1	1.758	110.71	0.0015	0.0595	0.001	6857	0.003	12.521	0.0005	0.001	0.0005	3200
2	2.516	121.91	0.0022	0.0785	0.002	6857	0.019	16.524	0.0007	0.002	0.0003	3550
3	5.948	157.59	0.0031	0.0975	0.0014	20571	0.105	20.520	0.0006	0.003	0.0002	7360

## Materials and Methods

### Materials required

Various chemicals including, sodium/Potassium hydroxide (NaOH/KOH), bleach (NaOCl), nitric acid (HNO_3_), Sodium Sulfide (Na_2_S), Magnesium sulfate (MgSO_4_), hydrochloric acid (HCl), Cadmium nitrate (Cd(NO_3_)_2_ .4H_2_O) and other reagents used were of AR grade. All the reagents and standards were prepared with Milli-Q water with a resistivity > 18.2 MΩ cm.

### Preparation of MgS@CNF

Agrowaste namely dried and powdered sugarcane bagasse was used to prepare cellulose nanofibers by the procedure reported elsewhere^23^. Briefly soxhlet extraction was initially performed on raw sugarcane bagasse for 6 h using organic mixture consisting of toluene and ethanol to remove the organic contaminants. Further, degradation of lignin was carried out using combination of bleach and acetic acid. Washing the product with distilled water and further treatment with KOH resulted in the removal of hemicelluloses moiety. This was followed by acid wash to remove residual alkali and air dried to obtain cellulose nanofibers (CNF).

Initially 2 g CNF was placed in a two neck Erlenmeyer flask with 200 mL of Milli Q water. This was followed by addition of 40 ml 0.2 M MgSO_4_.7H_2_O and the resultant mixture was stirred for 15 min. Further, around 20 ml of 0.4 M Na_2_S was added gradually using a syringe. The whole setup was kept in nitrogen atmosphere to avoid air oxidation. Continuous stirring for 30 min. ensured successful completion of the reaction and obtained product was washed and further heated for 200 °C in inert (N_2_) atmosphere for 2 h to obtain MgS@CNF. Obtained MgS@CNF was stored in dessicator until further use.

### Characterization of MgS@CNF

FEI Quanta 200 microscope was used for Energy Dispersive X-ray (EDX) and Field emission scanning electron microscopy (FE-SEM) images and after gold coating. Fourier Transform Infra-red (FTIR) spectra of Virgin and cadmium loaded MgS@CNF was obtained using Tensor 27 (Bruker, Germany). X-ray photoelectron spectroscopic measurements (XPS) were recorded with PHI 5000 Versa ProbII, FEI Inc. using a monochromatic Al Kα radiation (1486.6 eV). Deconvoluted spectra of Cd and Sulfur were produced using XPS PEAK 4.1 software with a Gaussian–Lorentzian sum function.

### Equilibrium adsorption experiments with CNF and MgS@CNF

Equilibrium adsorption experiments with both CNF and MgS@CNF in batch reactors. Equilibrations with both nanomaterials were performed for 16 h, maintaining the temperature at 25 °C in shaking incubator set to an rpm of 110. Filtration of samples were carried out after equilibration and analyzed for Cd(II) after suitable dilutions using inductively coupled plasma mass spectrometer (ICPMS). The experimental conditions performed were: dose of adsorbent: 0.5 g/l, pH: 5.5 for MgS@CNF and pH: 7.5 for CNF, equilibration time: 16 h, initial concentration of Cd(II): 10 mg/l, total aqueous volume: 20 ml. Equilibrium isotherm experiments were performed by varying initial Cd(II) concentrations (10 to 200 mg/l) for MgS@CNF and 10 to 100 mg/l for CNF. The amount of Cd(II) adsorbed by CNF and MgS@CNF were obtained from eqn (11):

11 $${q}_{e}=\frac{({C}_{i}-{C}_{e})XV}{W}$$

where *q*_*e*_ is the quantity of Cd(II) adsorbed by both the prepared adsorbents (mg/1), *C*_*i*_ and *C*_*e*_ are the initial and equilibrium Cd(II) concentration (mg/1), W is the mass of the sorbent used (g) V is final aqueous volume. For kinetic studies, determination of Cd(II) uptake was carried at defined time intervals.

## Conclusions

In the present study MgS doped cellulose nanofibres were prepared characterized and applied towards the removal of Cadmium from industrial wastewater. Cellulose nanofibers acted as a template for the effective dispersion of MgS. MgS acted as source of sulfide ions for complexing cadmium. The Langmuir monolayer adsorption capacity towards Cd(II) for MgS@CNF (333.3 mg/g) was found to be higher than virgin CNF (7.81 mg/g). Effective anchoring of MgS was evident from XPS and XRD studies. Detailed spectroscopic investigations revealed the formation of CdS which accounts for the very high adsorption capacity. Furthermore, the ability of MgS@CNF to remove Cd ions from industrial waste water is demonstrated. Finally, it could be postulated that similar to Cd, prepared adsorbent can find application in the removal of other heavy metal ions like Cr(VI), Hg(II), Pb(II), Cu(II) as well.

## Supplementary information

Performance of novel MgS doped cellulose nanofibres for Cd(II) removal from industrial effluent – mechanism and optimization
