# Supplementary material for: Performance of novel MgS doped cellulose nanofibres for Cd(II) removal from industrial effluent – mechanism and optimization
Source: Sci Rep. 2019 Sep 2;9:12639. doi: 10.1038/s41598-019-49076-2 (PMC6718681; doi:10.1038/s41598-019-49076-2)
Supplement: Supplementary file 1 — Performance of novel MgS doped cellulose nanofibres for Cd(II) removal from industrial effluent – mechanism and optimization [file 41598_2019_49076_MOESM1_ESM.docx]

**Performance of novel MgS doped cellulose nanofibres for Cd(II) removal from industrial effluent – mechanism and optimization**

Nalini Sankararamakrishnan, Rishabh Singh and Ila Srivastava

**Supporting Information**

**Fig. S1 XPS Survey Spectra of Cd-MgS@CNF**

**Table S1. Comparison of maximum adsorption capacities of various sorbents**

| Adsorbent | Adsorption Capacity | Reference |
| --- | --- | --- |
| Amine and Thiol fuctionalized activated carbon | 79.2 | 35 |
| Thiosemicarbazide modified polyacrylonitrile | 165.2 | 36 |
| Amine and thiolated multiwalled carbon nano tubes | 61.1 | 37 |
| Thiol modified silica gel |  | 38 |
| thiol-functional Cd^2+^-imprinted silica-based polymer | 62.9 | 39 |
| Chitosan - phenylthiourea | 120.0 | 40 |
| Oxalone modified Cellulose fibers | 66.3 | 41 |
| Multifunctional group modified Cellulose | 277.0 | 42 |
| Nano cellulose fibres | 9.7 | 43 |
| Sulfur chelating Cellulose | 54.71 | 44 |
| Magnetic sulfide-modified nanoscale zerovalent iron (S-nZVI) | 120 | 45 |
| Reactive sulfide modified nano iron | 105 | 46 |
| Cellulose nanofibers (CNF) | 7.81 | This Work |
| MgS@CNF | 333.33 | This Work |

**Table S2. Characteristics of Tannery waste water**

| **Parameters** | **Sample 1** | **Sample 2** | **Sample 3** |
| --- | --- | --- | --- |
| pH | 8.33 | 8.51 | 8.67 |
| Total Dissolved Solids | 1000 | 2400 | 2800 |
| Hardness | 430 | 560 | 800 |
| Sulfate | 136 | 1105 | 168 |
| Chemical Oxygen Demand | 6857 | 6857 | 20571 |
| Total Organic Carbon | 219.1 | 302.9 | 1277.5 |
| Total Nitrogen | 64.2 | 127.2 | 169.9 |
| Total Cr | 110.71 | 121.91 | 157.59 |
| Arsenic | 0.001 | 0.002 | 0.0014 |
| Cadmium | 1.758 | 2.516 | 5.948 |
| Lead | 0.0015 | 0.0022 | 0.0031 |
| Zinc | 0.0595 | 0.0785 | 0.0975 |
| Iron | 0.0313 | 0.0415 | 0.0648 |

All parameters except pH are in mg/l.

**References**

1. [Tang, N](https://www.ncbi.nlm.nih.gov/pubmed/?term=Tang%20N%5BAuthor%5D&cauthor=true&cauthor_uid=29710586)., [Niu C.G](https://www.ncbi.nlm.nih.gov/pubmed/?term=Niu%20CG%5BAuthor%5D&cauthor=true&cauthor_uid=29710586)., Li X.T., [Liang, C](https://www.ncbi.nlm.nih.gov/pubmed/?term=Liang%20C%5BAuthor%5D&cauthor=true&cauthor_uid=29710586)., [Guo, H](https://www.ncbi.nlm.nih.gov/pubmed/?term=Guo%20H%5BAuthor%5D&cauthor=true&cauthor_uid=29710586)., [Lin, L.S](https://www.ncbi.nlm.nih.gov/pubmed/?term=Lin%20LS%5BAuthor%5D&cauthor=true&cauthor_uid=29710586)., [Zheng, C.W](https://www.ncbi.nlm.nih.gov/pubmed/?term=Zheng%20CW%5BAuthor%5D&cauthor=true&cauthor_uid=29710586)., [Zeng, G.M](https://www.ncbi.nlm.nih.gov/pubmed/?term=Zeng%20GM%5BAuthor%5D&cauthor=true&cauthor_uid=29710586). Efficient removal of Cd^2+^ and Pb^2+^ from aqueous solution with amino- and thiol-functionalized activated carbon: Isotherm and kinetics modeling. [Sci Total Environ.](https://www.ncbi.nlm.nih.gov/pubmed/29710586), 635, 1331-1344 (2018).
2. S. Deng, P. Wang, G. Zhang, Y. Dou, Polyacrylonitrile-based fiber modified with thiosemicarbazide by microwave irradiation and its adsorption behavior for Cd(II) and Pb(II). J. Hazard. Mater., 307, 64-72(2016).
3. M. Hadavifar, N. Bahramifar, H. Younesi, M. Rastakhiz, Q. Li, J. Yu, *et al.* Removal of mercury(II) and cadmium(II) ions from synthetic wastewater by a newly synthesized amino and thiolated multi-walled carbon nanotubes. J. Taiwan Inst. Chem. Eng., 67 (2016), pp. 397-405
4. M. Najafi, R. Rostamian, A.A. Rafati Chemically modified silica gel with thiol group as an adsorbent for retention of some toxic soft metal ions from water and industrial effluent. Chem. Eng. J., 168 , 426-432(2011),
5. [Kong](https://pubs.rsc.org/en/results?searchtext=Author%3AQiaoping%20Kong), Q.,  [Xie](https://pubs.rsc.org/en/results?searchtext=Author%3ABinbin%20Xie), B.,  [S Preis](https://pubs.rsc.org/en/results?searchtext=Author%3ASergei%20Preis), S.,   [Hu](https://pubs.rsc.org/en/results?searchtext=Author%3AYun%20Hu), Y.,  [Wu](https://pubs.rsc.org/en/results?searchtext=Author%3AHaizhen%20Wu), H.,   and Wei, C., Adsorption of Cd^2+^ by an ion-imprinted thiol-functionalized polymer in competition with heavy metal ions and organic acids. RSCAdv.,8, 8950 - 8960 (2018).
6. [Monier](https://www.sciencedirect.com/science/article/pii/S0304389412000313#!), M., [Abdel-Latif](https://www.sciencedirect.com/science/article/pii/S0304389412000313#!), D.A. Preparation of cross-linked magnetic chitosan-phenylthiourea resin for adsorption of Hg(II), Cd(II) and Zn(II) ions from aqueous solutions, [Journal of Hazardous Materials](https://www.sciencedirect.com/science/journal/03043894),  [209–210](https://www.sciencedirect.com/science/journal/03043894/209/supp/C), 240-249 (2012)
7. Stephen, M.,Catherine, N.,Brenda, M.,Andrew, K.,Leslie, P. & Corrine, G. Oxolane-2, 5-dione modified electrospun cellulose nanofibers for heavy metals adsorption, Journal of hazardous materials. 192, 922-927 (2011).
8. [Chen, Q.](https://www.scopus.com/authid/detail.uri?origin=resultslist&authorId=56304816900&zone=), [Zheng, J.](https://www.scopus.com/authid/detail.uri?origin=resultslist&authorId=57195366322&zone=), [Wen, L.](https://www.scopus.com/authid/detail.uri?origin=resultslist&authorId=57191418738&zone=), [Yang, C.](https://www.scopus.com/authid/detail.uri?origin=resultslist&authorId=57207688034&zone=), &[Zhang, L.](https://www.scopus.com/authid/detail.uri?origin=resultslist&authorId=56178860600&zone=)[A multi-functional-group modified cellulose for enhanced heavy metal cadmium adsorption: Performance and quantum chemical mechanism](https://www.scopus.com/record/display.uri?eid=2-s2.0-85062713193&origin=resultslist&sort=plf-f&src=s&st1=cadmium+and+cellulose&st2=&sid=a27736761f797089dd75c11de5bde475&sot=b&sdt=b&sl=36&s=TITLE-ABS-KEY%28cadmium+and+cellulose%29&relpos=0&citeCnt=0&searchTerm=).Chemosphere. 224, 509-518 (2019).
9. Kardam, A., Raj, K.R., Srivastava, S.&Srivastava, M.M. Nanocellulose fibers for biosorption of cadmium, nickel, and lead ions from aqueous solution. Clean Technologies and Environmental Policy. 16, 385–393(2014).
10. [Zheng, L.](https://www.scopus.com/authid/detail.uri?origin=resultslist&authorId=55462969700&zone=), [Yang, Y.](https://www.scopus.com/authid/detail.uri?origin=resultslist&authorId=57205350637&zone=), [Meng, P.](https://www.scopus.com/authid/detail.uri?origin=resultslist&authorId=57023352500&zone=)&[Peng, D.](https://www.scopus.com/authid/detail.uri?origin=resultslist&authorId=55653450800&zone=) [Absorption of cadmium (II) via sulfur-chelating based cellulose: Characterization, isotherm models and their error analysis](https://www.scopus.com/record/display.uri?eid=2-s2.0-85059679994&origin=resultslist&sort=plf-f&src=s&st1=cadmium+and+cellulose&st2=&sid=a27736761f797089dd75c11de5bde475&sot=b&sdt=b&sl=36&s=TITLE-ABS-KEY%28cadmium+and+cellulose%29&relpos=6&citeCnt=1&searchTerm=). [Carbohydrate Polymers](https://www.scopus.com/sourceid/25801?origin=resultslist). 209, 38-50 (2019).
11. [Su, Y](https://www.ncbi.nlm.nih.gov/pubmed/?term=Su%20Y%5BAuthor%5D&cauthor=true&cauthor_uid=25706223)., [Adeleye, A.S](https://www.ncbi.nlm.nih.gov/pubmed/?term=Adeleye%20AS%5BAuthor%5D&cauthor=true&cauthor_uid=25706223)., [Keller, A.A](https://www.ncbi.nlm.nih.gov/pubmed/?term=Keller%20AA%5BAuthor%5D&cauthor=true&cauthor_uid=25706223)., [Huang, Y](https://www.ncbi.nlm.nih.gov/pubmed/?term=Huang%20Y%5BAuthor%5D&cauthor=true&cauthor_uid=25706223)., [Dai, C](https://www.ncbi.nlm.nih.gov/pubmed/?term=Dai%20C%5BAuthor%5D&cauthor=true&cauthor_uid=25706223)., [Zhou, X](https://www.ncbi.nlm.nih.gov/pubmed/?term=Zhou%20X%5BAuthor%5D&cauthor=true&cauthor_uid=25706223).&[Zhang, Y](https://www.ncbi.nlm.nih.gov/pubmed/?term=Zhang%20Y%5BAuthor%5D&cauthor=true&cauthor_uid=25706223). Magnetic sulfide-modified nanoscalezerovalent iron (S-nZVI) for dissolved metal ion removal. Water research. 74, 47-57(2015)
12. Su, Y.,Adeleye, A. S.,Huang, Y., Zhou, X., Keller, A.A. & Zhang, Y. Direct Synthesis of Novel and Reactive Sulfide-modified Nano Iron through Nanoparticle Seeding for Improved Cadmium- Contaminated Water Treatment. Scientific reports.6, 24358(2016).
